# Investigation of the Defect Structure of Congruent and Fe-Doped LiNbO_3_ Powders Synthesized by the Combustion Method

**DOI:** 10.3390/ma10040380

**Published:** 2017-04-02

**Authors:** You-Yun Li, Hao-Long Chen, Guo-Ju Chen, Chia-Liang Kuo, Ping-Hung Hsieh, Weng-Sing Hwang

**Affiliations:** 1 Department of Materials Science and Engineering, National Cheng Kung University, No. 1, University Road, Tainan 70101, Taiwan; li.youyun@gmail.com (Y.-Y.L.); jdempire1981@gmail.com (C.-L.K.); hann0613@gmail.com (P.-H.H.); 2 Department of Electronic Engineering, Kao Yuan University, No. 1821, Jhongshan Road, Lujhu District, Kaohsiung 82151, Taiwan; t11033@cc.kyu.edu.tw; 3 Department of Materials Science and Engineering, I-Shou University, No.1, Sec. 1, Syuecheng Road, Dashu District, Kaohsiung 84001, Taiwan; gjchen@isu.edu.tw

**Keywords:** Fe-doped lithium niobate, combustion method, defect structure, Rietveld refinement

## Abstract

Fe-doped LiNbO_3_ synthesized by the combustion method to seek new multiferroic materials exhibits room-temperature ferromagnetism, as reported in our previous work. In this work, the defect structure of congruent and Fe-doped LiNbO_3_ (0.57–3.3 mol %) powders was investigated in detail by several methods. The molar ratio of [Li]/([Li]+[Nb]) was determined by the Curie temperature (*T*_c_) via DSC. Two peaks of *T*_c_ were observed due to phase splitting, and the phase at lower *T*_c_ disappears as the Fe doping concentration increases. The coexistence of two different oxidation states of Fe ions in LiNbO_3_ was probed by XPS and UV-Vis spectroscopy. The Raman spectra exhibit displacements along the c axis of Li and Nb ions, and a deformation of the NbO_6_ framework owing to Fe doping. Several doping models were applied in the Rietveld refinement of powder X-ray diffraction collected by synchrotron radiation. The fitting by the Nb vacancy model leads to an improbably distorted structure of congruent LiNbO_3_. In Fe-doped LiNbO_3_, we conjecture that Li and Nb vacancies coexist in the lattice structure; Fe^+2^/Fe^+3^ ions are substituted for Li ions at the regular Li site and may push the anti-site Nb_Li_ ion back to the regular Nb site.

## 1. Introduction

The discovery of the single-phase multiferroic materials with high magnetoelectric susceptibility in room temperature for the intention of advanced functional devices fabrication and the investigation of the mechanism of the magnetoelectric coupling phenomena have been so far essential in the field of research [1,2,3]. Two major types of oxide candidates have been frequently investigated for their multiferroic properties. One is materials with magnetic ordering-induced multiferroic behavior, in which electric polarization is induced in a magnetically ordered state like complex spin structures, but a major limitation to further use is the low phase transformation temperature. The other type is the perovskite-type compound, in which the electric polarization and the magnetic coupling are donated by different ions in the lattice structure, and the Curie temperature is high enough for devices working at room temperature but the magnetoelectric coupling is small. However, only BiFeO_3_ has been widely investigated and was applied to the composite systems [4]. Nowadays, the limited choice of room-temperature single-phase multiferroics is one of the major challenges for scientists trying to discover new materials.

The well-known electro-optical and nonlinear optical material LiNbO_3_ has a distorted perovskite structure with high spontaneous polarization (*P*_s_ = 0.71 C/m^2^). LiNbO_3_ is an attractive host material to dope with a variety of elements for different kinds of applications. Transition metal ions like Mg, Zn, or In were shown to replace anti-site Nb_Li_ ion, hereafter labeled Nb* ion, to increase the damage resistance [5,6]. Fe, Cu, or Mn ions were proven to enhance the photorefractive effect [7,8]. Recently, attention was drawn to the ferromagnetism of undoped or doped LiNbO_3_ under room temperature [9,10,11,12]. 

LiNbO_3_ has a nonstoichiometric phase range due to Li deficiency, which brings on intrinsic defects like Nb* ion, Li vacancy, and Nb vacancy. Several research groups have debated various defect models [13,14,15,16,17] in LiNbO_3_. These defects have an intense impact on their physical property. The electronic coupling with ions and defects in LiNbO_3_ is stronger than in other ABO_3_ compounds; the charge transport in LiNbO_3_ is described in terms of electron self-trapping to form bound small polarons by Nb* ion or bound bipolarons by pairs of nearest-neighbor Nb ions [18]. It is more complicated in the doped one because the doping ions and intrinsic defects both participate in the charge transport, and the occupancy site and coupling mechanism would severely influence properties.

In Fe-doped LiNbO_3_, approaches towards the occupancy site and oxidation state of Fe ions have been studied in the past few decades to probe their relationship in the photo-voltaic effect. From electron-spin-resonance (EPR) measurements, Zhao et al. [19] showed evidence for Fe incorporation onto the regular Nb site, whereas Peterson et al. [20] conducted a field annealing experiment, indicating that Fe ions do not occupy the regular Nb site. In general, most believe that the Fe ion is incorporated onto the regular Li site. Lots of studies on X-ray absorption spectroscopy (XAS) [21,22,23] are in favor of the regular Li site. Furthermore, Gog et al. [24] employed the X-ray standing waves technique to conclude that Fe ions replace Li and Nb* ion at the regular Li site, but the micro-Raman analysis of Mignoni et al. [25] showed that the Fe ion goes on the regular Li site and pushes the Nb* ion back to the regular Nb site. Therefore, the detailed doping mechanism of Fe ions is still unclear.

Two oxidation state of Fe ions are found in LiNbO_3_ [20] so that the properties and the lattice structure are complicated. Fe^+2^ ion substitutes for Li^+^ [26] and then acts as an impurity center responsible for photorefractive effect. The mechanism of the optical intervalence transfer transition was interpreted by Schirmer et al. [27]. Small polarons are transported by thermally activated hopping from Fe^+2^ to Fe^+3^ and the neighboring Nb^+5^. Previous studies reported no evidence of interactions between Fe ion and the charge-compensating defect [26,28]. The quantitative characterization and modification of the Fe^+2^ content by annealing treatments are frequently investigated by the combination of optical absorption spectra, EPR measurement and standard samples for calibrations [28,29,30]. The local distortion induced by the self-trapping action related to the polaron formation was also investigated by some recent XRD results [31] and extended X-ray absorption fine structure (EXAFS) [32]; in these studies a larger lattice relaxation was measured when the Fe^+3^ changed to Fe^+2^. However, the concentration of Fe in these samples is relatively low; there was a lack of data on high doping samples to understand the deformation of the defect structure.

In our previous studies, the high Fe-doped LiNbO_3_ nanocrystals have been produced by the combustion method and their ferromagnetic properties at room temperature were investigated [33]. The combustion method is a simple and fast solution to the problems of long sintering time and high sintering temperature of LiNbO_3_ for solid-state processes [34,35,36]. In the whole reaction system, the initial mixture containing metal nitrates as oxidants and the amino acetic acid (glycine) as fuel are ignited and converted into the desired powders by self-sustaining fast combustion [37,38]. The self-sustaining reaction makes the mixture reach the high sintering temperature to form LiNbO_3_ nanocrystals. However, the sintering temperature in the self-sustaining reaction is unknown, so that both *X*_c_, affected by the high evaporating pressure of lithium, and the structure are unknown. 

In this study, congruent and Fe-doped LiNbO_3_ synthesized via the combustion method were analyzed in detail by differential scanning calorimetry (DSC), X-ray photoelectron spectroscopy (XPS), ultraviolet-visible spectroscopy (UV-Vis), Raman spectra, and Rietveld refinement of powder X-ray diffraction collected by synchrotron radiation. Several defect models were discussed and adopted in the structure refinement to find the proper interpretation of the defect structure of Fe-doped LiNbO_3_ of our samples.

## 2. Experimental Methods

Nanoscale LiNbO_3_ and Fe-doped LiNbO_3_ powders were synthesized via the combustion method. Lithium nitrate (LiNO_3_), Iron(III) nitrate nonahydrate (Fe(NO_3_)_3_ 9H_2_O), ammonium niobate(V) oxalate hydrate (C_4_H_4_NNbO_9_ nH_2_O), and glycine (C_2_H_5_NO_2_) with at least 99.4% purity (all supplied by Sigma-Aldrich Chemical Company, Inc., St. Louis, MO, USA) were used as starting materials. LiNbO_3_ powders were prepared at a non-stoichiometric molar ratio, *X*_c_ = 41%–43% (hereinafter denoted as LN41–LN43), and LiNbO_3_ doped with *x*% Fe (where *x* = 0.5, 1, 2, 3, 5) was synthesized as *x*% mole Fe +100% mole LN43 (hereinafter denoted by FLN-A-43, FLN-B-43, FLN-C-43, FLN-D-43, and FLN-E-43, respectively). The 0.3 M stoichiometric aqueous nitrate solutions were bathed until a viscous precursor formed. The viscous precursor was conducted in an alumina crucible and then calcined at 600 °C for 1 h under oxygen atmosphere with a heating rate of 5 °C/min.

Since LiNbO_3_ contains the Li element and is hard to dissolve in acid solution, the analysis of the composition is difficult. In this work, we derive Li concentrations of these samples from the relationship between *X*_c_ and *T*_c_. *T*_c_ is linearly dependent on the Li_2_O content; it is roughly 150 °C below the melting point of stoichiometric LiNbO_3_ (1480 K), which provides sufficient precision for composition measurements (0.1–0.3 mol %) [39,40]. The *T*_c_ of these samples was measured by using the differential scanning calorimetry (STA 449 F3, Netzsch, Selb, Germany) in a high-quality nitrogen atmosphere (99.99%) and at a heating rate of 5 °C/min. The Fe/Nb ratio of Fe-doped samples was determined by the electron probe microanalyzer (JXA-8800M, JEOL, Tokyo, Japan). 

For X-ray photoelectron spectra (XPS) studies, the sample powders were pressed into disks. XPS measurements were performed using a PHI Quantera SXM/AES 650 Auger Electron Spectrometer (ULVAC-PHI, Kanagawa, Japan) equipped with a hemispherical electron analyzer and a scanning monochromatic Al Kα source (1486.6 eV). The samples were depressurized overnight before the analysis and the base pressure in the analysis chamber was 10^−9^ Torr. A low-energy Ar^+^ ion gun was used to neutralize the samples and the take-off angle for photoelectrons was fixed at 45°. Survey spectra were recorded with 1.0 eV step and 280 eV analyzer pass energy and the high resolution regions were recorded with 0.1 eV step and 55 eV pass energy. The photoelectron lines of Nb 3d, O 1s, and Fe 2p were recorded and the C 1s line of contaminating carbon with the binding energy of 284.4 eV [41] was used as a reference for calibration. The ultraviolet-visible (UV-Vis) absorption spectra of the samples were examined in the 300–700 nm wavelength range by a UV/Vis/NIR spectrophotometer (U-4100, HITACHI, Tokyo, Japan). The Raman spectra for undoped LiNbO_3_ (LN41 to LN42) were also collected to compare with those from the previous study on Fe-doped LiNbO_3_ [33]; both sets were recorded on a LabRAM HR Raman spectrometer (Horiba Jobin Yvon, Lille, France) with a 785 nm wavelength diode laser in the extended range of 100–1000 cm^−1^. The powder X-ray diffraction (PXRD) experiment of the samples for the structure refinement was carried out using high-energy synchrotron radiation at the BL12B2 beamline of SPring-8, Hyogo Prefecture, Japan. The powder diffraction patterns were recorded at the wavelength of 0.68898 Å (18 KeV) and calibrated by using the Bragg peaks of NIST LaB6 standard. Powdered samples were loaded into a 0.5-mm capillary for uniform absorption and the distance from sample to CCD detector (MS225, Rayonix, Evanston, IL, USA) was about 150 mm, in which the angular resolution is 0.021 degrees in two theta. The X-ray exposure time was 30 s during data collection and 2D diffraction patterns were converted to 1D profiles with cake-type integration by GSAS-II program for structure refinement. 

## 3. Results and Discussion

Several intrinsic defect models have been proposed in the literature to describe congruent LiNbO_3_. The Li deficiency in the lattice contributes to Nb* ions. In the Li vacancy model [15], the charge neutrality is guaranteed by the creation of Li vacancies. For one Nb* ion, there are four Li vacancies, hereafter denoted as VLi, so that the corresponding chemical formula can be written as:

[Li_1-5*x*_Nb*_x_VLi_4*x*_][Nb]O_3_. (1)

In the Nb vacancy model [14], Nb vacancies, hereafter denoted as VNb, are created to maintain the charge neutrality. Four VNb are needed for five Nb* ions, and the corresponding chemical formula can be written as:

[Li_1-5*x*_Nb*_5*x*_][Nb_1-4*x*_VNb_4*x*_]O_3_. (2)

The Li vacancy model was supported by NMR results [42] and the structure refinement via means of X-ray diffraction and neutron diffraction. In the past decades, Li vacancy model was recognized as the proper model to interpret the defect structure of LiNbO_3_. However, recently several mixed-vacancy models that combine the Li vacancy model and Nb vacancy model were discussed. Abdi et al. [13] suggested that VLi and VNb coexist in the lattice by comparison with the Raman spectra and electro-optical data. They preferred a mixed-vacancy model based upon Nb vacancy model (for *X*_c_ from 48.5% to 48.9%). For *X*_c_ beyond 48.9%, the defect model would follow the way of the Li vacancy model. In their mixed-vacancy model, VLi would increase and VNb would disappear as increasing the incorporation of Li into the lattice. Li et al. [16,17] have studied the charge transition levels of the intrinsic point defects and the formation energies of these defect clusters from first principle method. In addition to Li and Nb vacancy model, they proposed a mixed-vacancy model to do the calculation. Their result indicated that the total formation energy is in favor of Li vacancy model, while the Nb vacancy model has the lowest formation energy per single point defect, and they noted that VNb may occur in the strongly n-type sample. Therefore, the mixed-vacancy model could describe the defect structure of a certain type of metal-doped LiNbO_3_. The mixed-vacancy model proposed by Li et al. can be rewritten as:

[Li_1-5*x*_Nb*_2*x*_ VLi_3*x*_][Nb_1-*x*_VNb*_x_*]O_3_. (3)

In our work, Li vacancy model, Nb vacancy model, and the mixed-vacancy model proposed by Li et al. were adopted for the structure refinement.

The Li concentration of our samples was determined by the equation of *T*_c_ and *X*_c_. Iyi et al. [15,40] described the linear dependence of *T*_c_ on Li concertation by the following equation:

*X*_c_ = 0.02546*T*_c_ (°C) + 19.39, (4)

where *X*_c_ is equal to the molar ratio of [Li]/([Li]+[Nb]) and denotes as the mol % of Li of the compound; *T*_c_ is in Centigrade.

**Figure 1:**
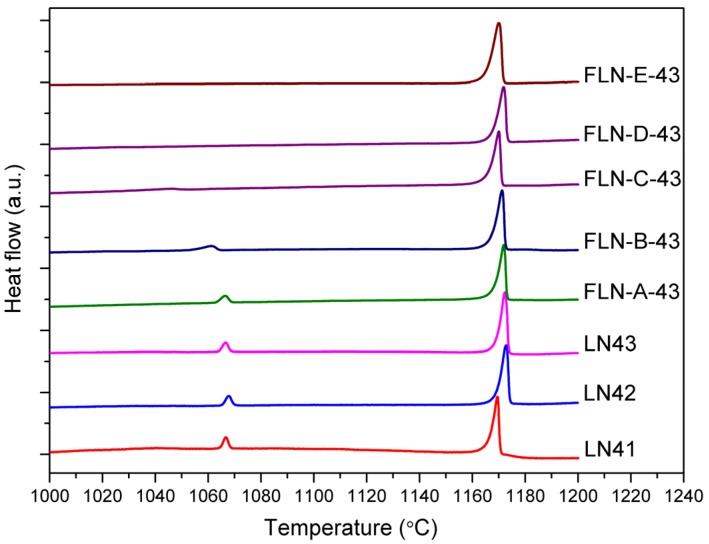
DSC curves of congruent LiNbO_3_ (LN41 to LN43) and Fe-doped LiNbO_3_ (FLN-*x*-43, *x* = A to E).

Figure 1 displays the DSC curve of congruent LiNbO_3_ and Fe-doped LiNbO_3_. The value of each *T*_c_ peak and the corresponding *X*_c_ by Equation (4) are shown in Table 1. Fe/Nb ratio of Fe-doped LiNbO_3_ is shown in Table 2. Figure 1 demonstrates that there are two phase transition peaks in the DSC curve of congruent LiNbO_3_, which correspond to *X*_c_ = 46.55% and 49.24% in LN43. Moreover, the intensity of the higher *T*_c_ was much stronger than the lower one. The existence of two peaks coincides with the observation of phase splitting in the previous report [36], where Li_0.91_NbO_3_ and LiNbO_3_ structures were formed to reduce the lattice distortion in the calcining process. There is a deviation between the calculated *X*_c_ and the stoichiometry in the experiment procedure that could be attributed to the indeterminate amount of water of crystallization in the ammonium niobate (V) oxalate hydrate. We have also observed that there was no apparent change in the *T*_c_ of congruent LiNbO_3_ with different stoichiometry. It seems that the combustion method is insensitive to the stoichiometry of LiNbO_3_ and has a wide tolerance for the chemical composition error. The Fe-doped LiNbO_3_ with relatively low concentration below 1 mol % like FLN-A-43 and FLN-B-43, also has two *T*_c_ peaks and the values are similar to those of undoped ones. The lower *T*_c_ peak disappears in Fe-doped LiNbO_3_ with a concentration above 1 mol %, such as FLN-*x*-43 (*x* = C, D, E). Although Equation (4) is applied for undoped LiNbO_3_, it may still imply that doping by Fe eliminates the phase that contains relatively more defects and hence renders the crystal structure stable.

**Figure 2:**
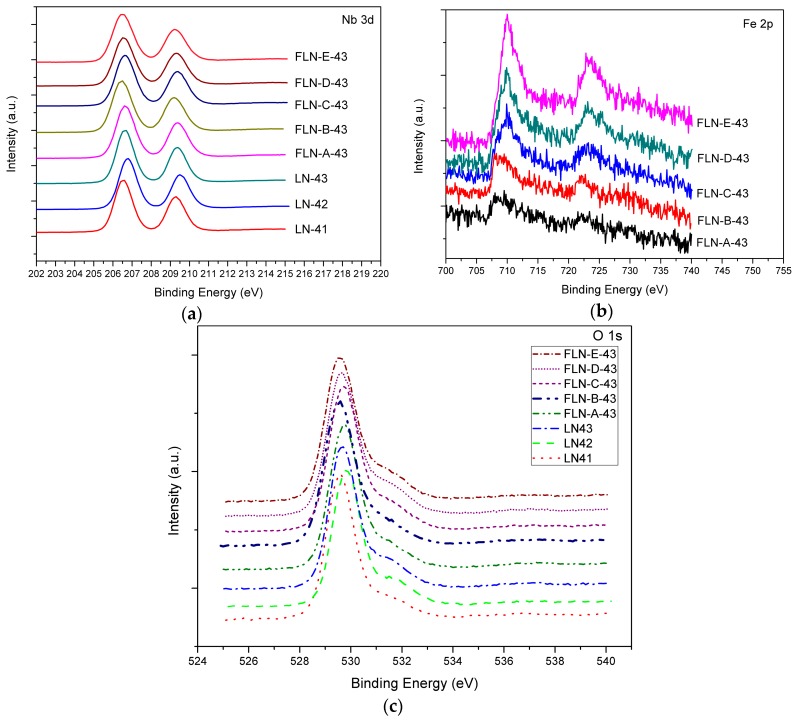
(**a**) Nb 3d peaks; (**b**) Fe 3d peaks and (**c**) O 1s peaks on the XPS spectra of congruent LiNbO_3_ (LN41 to LN43) and Fe-doped LiNbO_3_ (FLN-*x*-43, *x* = A to E).

From the XPS analysis, we observed the chemical state of congruent and Fe-doped LiNbO_3_ by checking the changes of the charge value of the Nb and Fe ions as shown in Figure 2. In general, the common oxidation states of Nb are either +5, as in Nb_2_O_5_, where the biding energies of 3d_5/2_ and 3d_3/2_ were 207.5 and 210.2 eV [43], or +4, as in NbO_2_, where the biding energies of 3d_5/2_ were 205.9 eV [44], and the energy gap is about 1.6 eV from the +5 to the +4 state. P. Steiner et al [45] investigated the chemical state of stoichiometric LiNbO_3_ with biding energies of 3d_5/2_ located at 207.1 eV. Figure 2a is the Nb 3d core level spectrum, the shape of each peak is symmetrical so that it could not be separated into two peaks, and the displacement of the peaks is too small to detect, which may be due to the relatively minor concentration ratio of Nb* to Nb. The biding energies for Nb 3d_5/2_ and 3d_3/2_ in our study were 206.6 and 209.5 eV, respectively. This is reasonable for congruent LiNbO_3_ because these values are between +4 state and the values for stoichiometric LiNbO_3_, and even closer to the latter. Figure 2b displays the Fe 2p core level spectrum; the biding energies for Fe 2p_3/2_ and 2p_1/2_ were 710 and 723 eV, which means the oxidation state of Fe was a mixture of divalent and trivalent ions [46]. The O 1s core level spectrum is shown in Figure 2c. The main peak is located at 529.6 eV, which is characteristic of oxygen in the metal oxide, and a small peak emerges near the main peak and is located at 531.6 eV, which is characteristic of oxygen in hydroxyl groups. The OH bond in LiNbO_3_ has been studied in FTIR spectra. The proton was assumed to be incorporated into the crystal defects, while LiNbO_3_ was grown in a humid atmosphere by the Czochralski method [47]. We conjecture that the OH bond exists in XPS by the same reason. The proton comes from the air in the combustion process and the OH bond is the combination of the proton with the O ion near the vacancy defect. 

**Figure 3:**
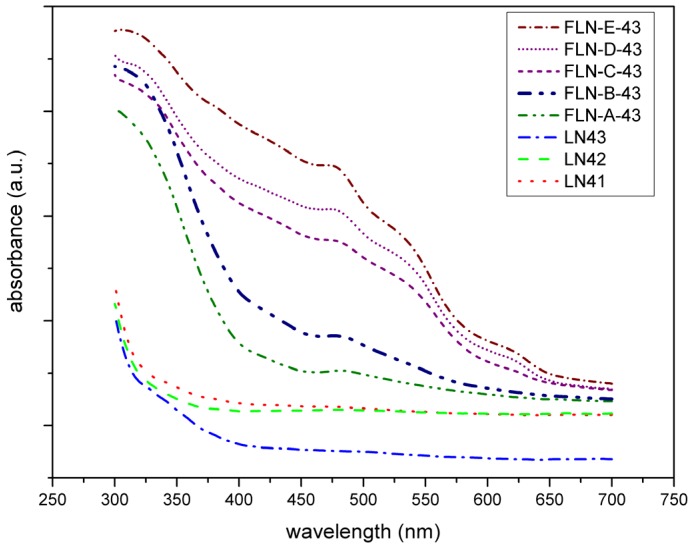
UV-Vis absorption spectra of congruent LiNbO_3_ (LN41 to LN43) and Fe-doped LiNbO_3_ (FLN-*x*-43, *x* = A to E).

Figure 3 exhibits the UV-Vis absorption spectra of our samples. It visualizes the absorption peak at 480 nm in each spectrum of Fe-doped LiNbO_3_ and the intensity increases as the doping concentration increases, which is proportional to Fe^+2^ concentration. These features coincide with previous reports [26,27,28,29,30,48,49]. The peak at 480 nm is assigned to the excitation of the electrons of Fe^2+^ to the conduction band by the photon and the intervalence transfer between Fe^+2^ and Nb^+5^ at d band [27]. Furthermore, from the view of the trend of absorption edge shifting, we can see that the spectra of congruent LiNbO_3_ were similar, but for Fe-doped ones, the slope of the curve near the absorption edge became steeper and the intercept point shifts to the longer wavelengths. The position of the absorption edge is related to the transition energy of the valence electron from 2p orbits of O^2−^ to 4d orbits of Nb^5+^ in LiNbO_3_. Doping with ions of high polarization ability enhances the deformation degree of O^2−^ 2p orbits, and therefore decreases the width of forbidden band and the transition energy, resulting in the redshift of the absorption edge [50]. The polarization ability values of the Fe^3+^, Li^+^, and Nb^5+^ ions are 55.32, 2.49, and 58.51, respectively [51]. Therefore, the Fe ion substitutes for the Li ion at the regular Li site. The shift is greater with increasing doping concentration, the same as in the examined literature [26,29].

**Figure 4:**
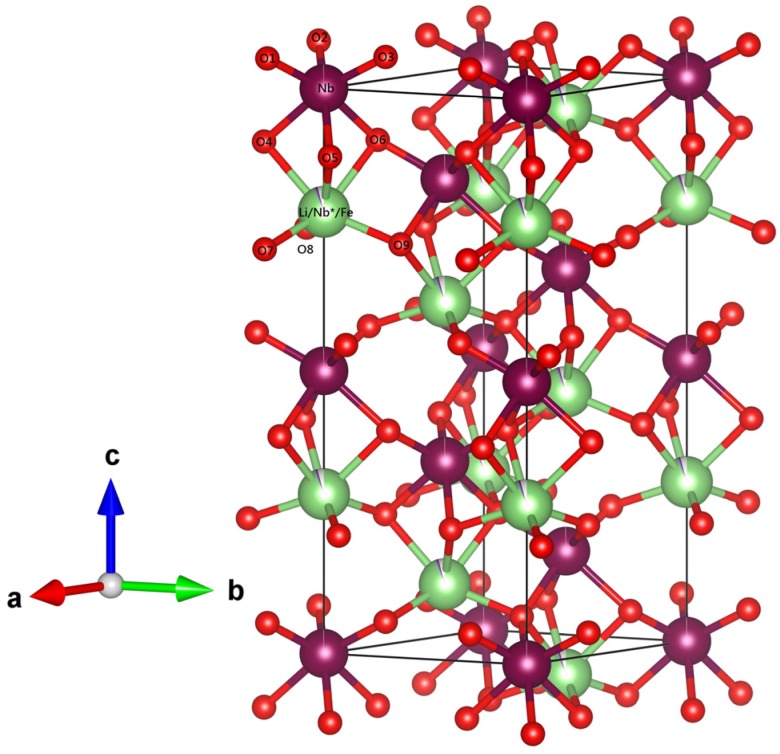
Atomic structure of Fe-doped LiNbO_3_; Li ion (in green), Fe ion (in blue), Nb ion (in purple), O ion (in red).

In order to understand the structure of LiNbO_3_ synthesized via the combustion method, we discuss the Raman spectra of the series of congruent LiNbO_3_, and then compare them to the Raman data of Fe-doped LiNbO_3_ from the previous study [33]. LiNbO_3_ belongs to the 3m point group and R3c space group. LiNbO_3_ structure could be described as an array of distorted octahedral oxygen cages (LiO_6_, NbO_6_, and ʋO_6_, where ʋ is a vacancy) sharing faces along the c axis. The octahedral interstitials are filled in the order Nb^5+^ , Li^+^, ʋ, Nb^5+^, …, which shift from the symmetrical position of the octahedron site along the c axis, as shown in Figure 4. Generally, doping ions would affect the deformation of these oxygen octahedra. Therefore, Raman spectrum is suggested to investigate the defect structure of the doped LiNbO_3_.

**Figure 5:**
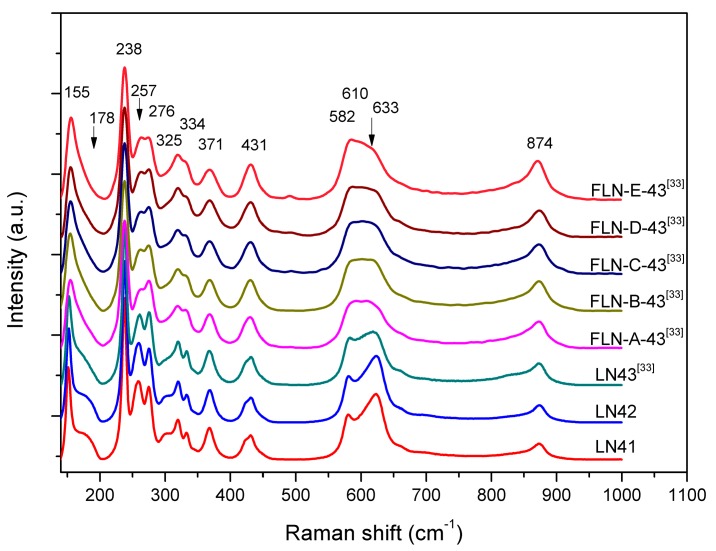
Raman spectra of the nonstoichiometric LiNbO_3_ (LN41, LN42, and LN43 [33]) and Fe-doped LiNbO_3_ (FLN-*x*-43, *x* = A to E) [33] at 145–1000 cm^−1^.

Consequently, 18 vibration modes are expected in the Raman spectra of LiNbO_3_ crystal and decomposed into 4A1 + 9E + 5A2, where A1 and E are both Raman and IR active while A2 are Raman inactive. The ionic motions associated with A1 (TO) modes are simpler and all four TO modes are correctly predicted from theory and detected in the experiment. However, the vibrational E modes are relatively complicated and none of the configurations allows for the detection of all 9 E TO modes. The assignment of the two missing modes is inconsistent in the literature [52,53,54,55,56]. Because our samples are powders, the scattering geometry was not specified for the detection. The results shown in Figure 5 confirm the assignment of Raman spectra by Repelin et al. [54], and are also similar to the Raman spectra of Fe-doped LiNbO_3_ reported by Mignoni et al. [25].

The frequencies in the 270–400 cm^−1^ are influenced by Li cation displacements. There is no evident change from LN41 to LN42, but the peaks are sharper in the undoped LiNbO_3_ than in Fe-doped LiNbO_3_, indicating that doping with Fe causes the deformation of the NbO_6_ framework and the distortion of the crystal. As shown in Figure 5, the peaks of 257 cm^−1^ shifts to 262 cm^−1^ and the peak intensity decreases. This observation is similar to the report by Mignoni et al. [25]. The peaks of 257 cm^−1^ are A1 (TO1) mode. According to calculations of Caciuc et al. [52], the A1 (TO1) mode corresponds to a vibration along the c axis of Nb ion, while Repelin et al. [54] regarded it as related to the deformation of NbO_6_ framework, mainly by oxygen atom shifts and small shifts of Li atoms. Mignoni et al. adaapted the interpretation of Caciuc et al. and speculated that Fe ions are incorporated onto the regular Li site and also push Nb* ions back to the regular Nb site, causing the vibration of Nb along the c axis. However, it should be noted that decreasing the concentration of Nb* at the regular Li site means decreases in the photoconductivity, which is typical for Mg-doped LiNbO_3_, not for Fe-doped. On the other hand, the idea that an Fe ion incorporated onto the regular Nb site could cause the vibration of Nb directly was disproved by most previous studies, as mentioned above. Therefore, we would take this dispute into consideration while doing the structure refinement in the later sections.

The A1 (TO2) mode is at 276 cm^−1^, called the Li-O stretching mode, and is strong related to the vibration of Li ions along the c axis. As shown in Figure 5, the peak of 276 cm^−1^ shifts to 274 cm^−1^ and the peak intensity decreases. In the study of Mg-doped LiNbO_3_ by Sidorov et al. [57], the peaks at 257 cm^−1^ and 276 cm^−1^ broaden and merge together when the doping concentration increases. In our study, a similar result was observed, which means that those Fe ions go on the regular Li site and strongly influence the displacement and bonding of Li-O in the lattice. The peaks at 334 cm^−1^ and 633 cm^−1^ correspond to A1 (TO3) and A1 (TO4) mode, both related to the rotation and stretching of the NbO_6_ framework caused by the shift of oxygen anions. It can be seen from Figure 5 that as the peaks at 334 cm^‑1^ and 633 cm^−1^ shift towards low frequencies, the peak shape becomes broader and the intensity decreases with the increase in Fe-doping concentration. This reveals that doping with Fe causes the deformation of the whole oxygen octahedral.

The deformation of the NbO_6_ framework could also be probed by the observation of E (TO1), E (TO2), E (TO8), and E (TO9) modes. It could be seen that the peak at 155 cm^−1^, corresponding to the E (TO1) mode, shifts towards higher frequencies. The E (TO1) mode is related to the deformation of the NbO_6_ framework on the *xy* plane, which implies that doping with Fe causes changes in the lattice parameter a. The peak at 178 cm^−1^, the disputed missing E mode, which corresponds to E (TO2), is observed in undoped LiNbO_3_ and disappears as the doping concentration increases in Figure 4. Repelin et al. [54] pointed out that it is related to the O–Nb–O bending. E-mode-related O–Nb–O stretching is also visible in the E (TO8) and E (TO9) modes, and at the peaks at 582 cm^−1^ and 610 cm^−1^, where there is a missing E mode. Figure 4 shows that there has been an obvious change in the peak shape, shift, and intensity of the peak from 582 cm^−1^ to 633 cm^−1^. Therefore, we infer that replacing Li ions by Fe gives rise to the O–Nb–O stretching and bending in the different direction and finally results in the deformation of the NbO_6_ framework.

**Figure 6:**
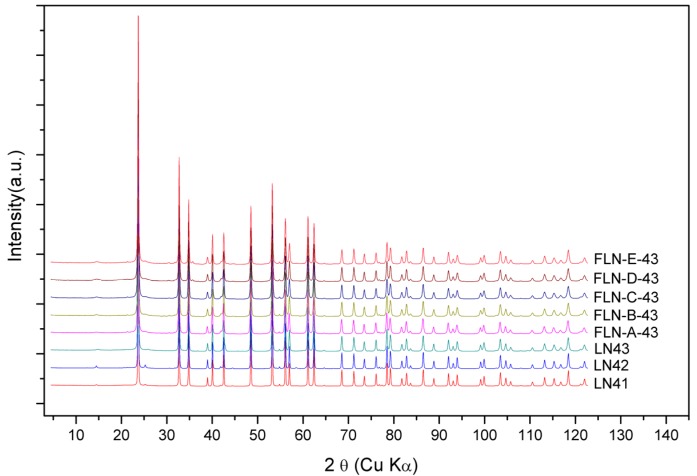
The powder diffraction patterns of congruent LiNbO_3_ and Fe-doped LiNbO_3_ collected by using synchrotron radiation (demonstrated by wavelength of Cu Kα).

The powder diffraction patterns of congruent LiNbO_3_ and Fe-doped LiNbO_3_ collected by synchrotron radiation were shown in Figure 6, which is demonstrated with the wavelength of Cu Kα. The powder diffraction data were analyzed to refine the defect structure of our samples via the Rietveld refinement program GSAS. As mentioned above, the Li vacancy model, the Nb vacancy model, and the mixed-vacancy model proposed by Li et al. would be adopted into the structure refinement in our work. Based on the data of DSC, shown in Table 1 and Figure 1, the peak intensity at the higher *T*_c_ is stronger than at the lower *T*_c_ and the phase of the lower *T*_c_ disappears in high Fe-doped samples. This means that the phase of the higher *T*_c_ is more stable than that of the lower *T*_c._ Thus, the *X*_c_ calculated from the higher *T*_c_ peak was adopted to refine the structure of LN43.

**Figure 7:**
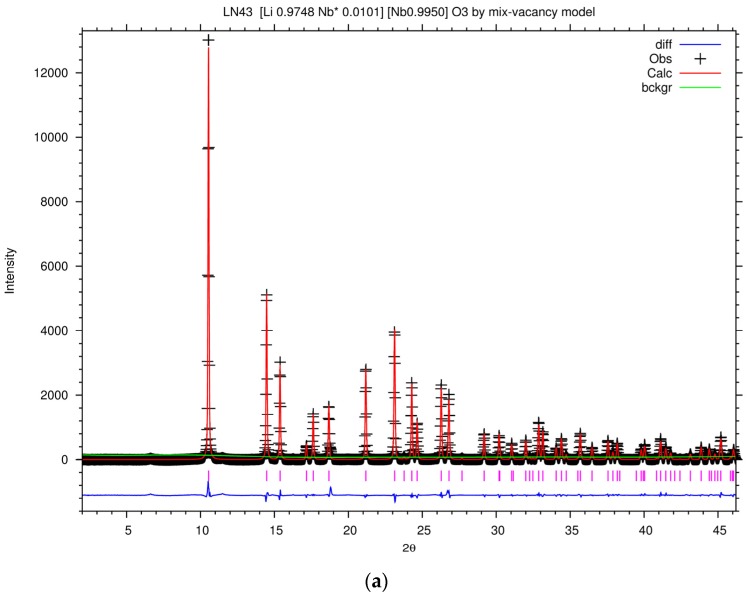

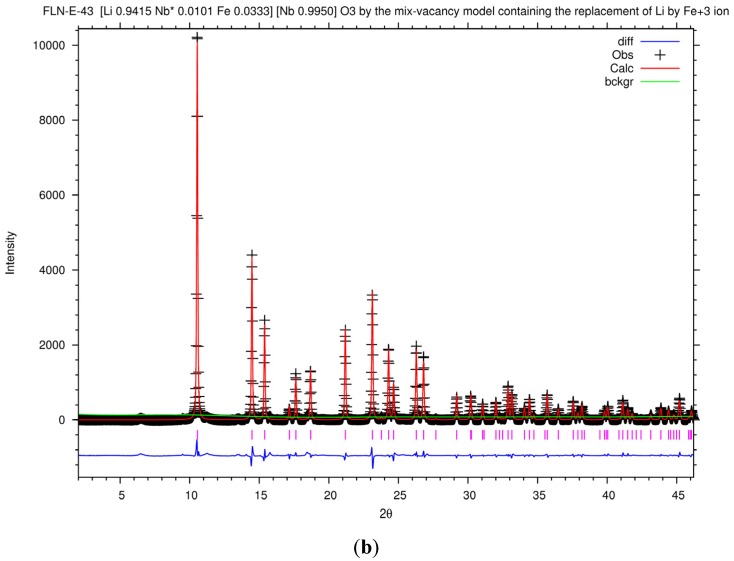
The Rietveld plot of the PXRD patterns of (**a**) LN43 by the mixed-vacancy model; (**b**) FLN-E-43 by the mixed-vacancy model containing the replacement of Li ion by Fe^+3^ ion; (+) observed data, (red line) calculated intensity, (green line) background, (magenta bars) the expected positions of Bragg reflections, and (blue line) difference curve, respectively.

Figure 7a shows the Rietveld plot of the PXRD patterns of LN43 by the mixed-vacancy model, and the Rietveld fit results of the lattice and agreement parameters of LN43 in different models are listed in Table 3. The goodness of fit parameters for the fit, like χ^2^, *R*_wp_, *R*_p_, and *R*_(f_^2^_)_ are acceptable. However, some of the isotropic displacement parameters, Uiso factors, are implausible. Uiso factor is related to the deformation of the electron density around the atom due to chemical bonding and affected by the absorption and other instrumental effects [58]. For tightly bound atoms in a metal oxide, a typical value of Uiso varies from 0.0063 to 0.038 Å^2^. Usually, the range of Uiso_Li/Nb*_ in LiNbO_3_ is around 0.01 to 0.02 [15,59,60,61]. It could be seen that the value of Uiso_Li/Nb*_ in the Nb vacancy model is too high and in the Li vacancy is is below 0.01. The value of Uiso_o_ in the mixed-vacancy and Li vacancy models is negative, which is also implausible. During the process of modifying models, it was found that the value of Uiso_o_ would increase when increasing the occupancy of O or decreasing the occupancy of Nb, and the influence of the occupancy of Nb is stronger than O. Increasing the occupancy of O would also raise the value of Uiso_Li/Nb*_ by a small amount. The small value of Uiso_o_ in the mixed-vacancy and Li vacancy models may be owing to improper absorption during the experiment or the existence of a second phase like Li_3_NbO_4_. However, decreasing the occupancy of Nb would inevitably result in an implausibly large value of Uiso_Li/Nb*_. In the Nb vacancy model, the occupancy of Nb is lower than in the other two models, which contributes to a positive value of Uiso_o_ and an unacceptably high Uiso_Li/Nb*_.

It is necessary to verify the rationality of these models in detail, thus, we investigate the refined atomic position, bond lengths, and bond angles of LN43, listed in Table 4 and Table 5. The corresponding position of O, Li, and Nb ions are represented in Figure 4. Comparing with previous studies on neutron and synchrotron x-ray powder diffraction of LiNbO_3_ [15,59,60,62], there is an obvious disparity in terms of the refined position of Li, Li–O bond lengths, and O–Li–O bond angles of LN43 in the Nb vacancy model. This reveals that applying the Nb vacancy model in the refinement leads to an improbably distorted structure. On the other hand, the refined bond length and bond angles in the mixed-vacancy model and Li vacancy model are similar to the literature reports. The difference between the formulae calculated by the mixed-vacancy model and the Li vacancy model is the occupancy of Nb* in LiNbO_3_. Higher occupancy of Nb* in the regular Li site brings out the higher Uiso_Li/Nb*_ and causes the lattice relaxation. Actually, the difference of lattice parameters, bond lengths, and bond angles between these two models is unremarkable. The distance of Li-vacancy-Nb, indicating the shift of the Li ion along the c axis, is longer, whereas the bond lengths of Li–Nb along the c axis and the average bond length of Li–O are a little shorter in the mixed-vacancy model than in the Li vacancy model. O–Li–O bond angles in the mixed-vacancy model stretch more than in the Li vacancy model. In our study, the mixed-vacancy model and Li vacancy model are both appropriate for interpreting the structures of congruent LiNbO_3_ synthesized by the combustion method.

The oxidation state of Fe is trivalent in Equations (5) to (8) and divalent in Equation (9). The charge-compensating defects for Fe ions replacing Li ions were excluded in our study. Equation (5) is rewritten from the mixed-vacancy model and one Fe^+3^ ion replaces one Li^+^ ion; Equation (6) is rewritten from the mixed-vacancy model including the replacement of Li^+^ ions and the item of pushed-back Nb* ions, and Equation (8) is rewritten from the mixed-vacancy model containing the replacement of both Li and Nb* ions as two Fe^+3^ ions replace one Li^+^ ion and one Nb* ion. Equations (7) and (9) are rewritten from Li vacancy model based on the replacement of Li^+^ ions only. In Equations (5) to (9), the value of x is the same as in LN43, calculated from X_c_ in DSC data; y means the concentration of Fe ions, which is calculated from the Fe/Nb ratio EPMA data listed in Table 2; α means the ratio of pushed-back Nb* ions over Fe ions.

The formulae for Fe-doped LiNbO_3_ are rewritten based on the mixed-vacancy model and Li vacancy model. According to the above discussion of XPS+, UV-Vis and Raman spectra, the proposed formulae would be based on the assumption that Fe ions are only incorporated onto the regular Li site. The incorporation of the Fe ion onto the regular Li site could be described as the substitution of Li ion only or Li and Nb* ion both. Since the oxidation state of Fe needs to be considered and the reported ratio of Fe^+2^/Fe^+3^ in as-grown Fe-doped crystal is about 0.045–0.17 [30,63], the discussion will focus mainly on the model with Fe^+3^ ions. The dispute about the Nb* ions pushed back to the regular Nb site was also taken into consideration. The formulae for Fe-doped LiNbO_3_ were listed below:

The mixed-vacancy model containing the replacement of Li ion by Fe^+3^ ion:

[Li_1-5*x*-*y*_Nb*_2*x*_VLi_3*x*_Fe*_y_*][Nb_1-*x*_VNb*_x_*]O_3_. (5)

The mixed-vacancy model containing the replacement of Li by Fe^+3^ ion and pushed-back Nb* ions:

[Li_1-5*x*-*y*_Nb*_2*x*-*ay*_VLi_3*x*+*αy*_Fe*_y_*][Nb_1-*x*+_*_αy_*VNb*_x_*_-_*_αy_*]O_3_. (6)

Li vacancy model containing the replacement of Li ion by Fe^+3^ ion:

[Li_1-5*x*-*y*_Nb*_x_VLi_4*x*_Fe*_y_*][Nb]O_3_. (7)

The mixed-vacancy model containing the replacement both of Li and Nb* ion by Fe^+3^ ion:

[Li_1-5*x*-*y*_Nb*_2*x*-*y*_VLi_3*x*_Fe_2*y*_][Nb_1-*x*_VNb*_x_*]O_3_. (8)

Li vacancy model containing the replacement of Li ion by Fe^+2^ ion:

[Li_1-5*x*-*y*_Nb*_x_VLi_4*x*_Fe*_y_*][Nb]O_3_. (9)

Looking at Equation (6), these pushed-back Nb* ions cause an increase of VLi and a decrease of VNb. If *αy* is equal to *x*, VNb would all be eliminated and Equation (6) is equal to Equation (7). Since α is undetermined, an assumption needs be made in our study in order to verify the rationality of the speculation on the pushed-back Nb* ions. The highest doping concentration in our samples is 3.3 mol % in FLN-E-43. If VNb is expelled in FLN-E-43, the value of α would equal 0.1514 and the formula of FLN-E-43 calculated by Equation (6) would be the same as by Equation (7). Thus, an arbitrary value of α as 0.0757 is applied in Equation (6) to investigate the defect structure containing parts of pushed-back Nb* ions and VNb.

Figure 7b shows the Rietveld plot of the PXRD patterns of FLN-E-43 by the mixed-vacancy model containing the replacement of Li ion by an Fe^+3^ ion. The Rietveld fit results of the lattice and agreement parameters of FLN-E-43 in different models are listed in Table 3 and the corresponding refined atomic position, bond lengths, and bond angles are listed in Table 4 and Table 6. Although the indicators of good of fitness show a good fit result, the value of Uiso_Li/Nb*/Fe_ in the model by Equation (8) is too low to be considered acceptable. The occupancy of Nb* in the regular Li site strongly affects Uiso_Li/Nb*/Fe_ and the replacement of Nb* ion would severely decrease the values of Uiso_Li/Nb*/Fe_. We deduce that the mixed-vacancy model containing the replacement of Nb* ions by Fe^+3^ ions is inappropriate. The major difference between models in terms of Equations (5)–(7) under the same doping concentration of Fe is the concentration of pushed-back Nb* ion. The value of Uiso_Li/Nb*/Fe_ in the model by Equation (5) is 0.0226, which is beyond the reported range of LiNbO_3_. We inferred that the mixed-vacancy model containing items of the pushed-back Nb* ions and Li vacancy model is more reasonable for describing the defect structure of Fe-doped LiNbO_3_ than the mixed-vacancy model without items of the pushed-back Nb* ions.

From Table 3, it shows that doping Fe ion in LiNbO_3_ causes lattice shrinkage. The position of Li in the c axis is reported to be shifting upward by about 0.1 Å in the data of Sanson et al. [32]. Since the location of Li ion is relative to the Nb ion along the c axis, a slight upward shift of Li ions could be observed from the increasing distance of Li-vacancy-Nb. Comparing the Li vacancy model and the model using Equation (7), the increment of the distance of Li-vacancy-Nb is around 0.042 Å in our study. The reported value of Fe^+3^–O bond distances is about 1.98 to 2.07 Å, which is smaller than the bond length of Li–O [32]. The bond lengths and bond angles of Li–O and Fe–O in Fe-doped LiNbO_3_ are under hybrid calculation in our refinement and denoted as Li/Fe–O in Table 6. The bond lengths of Li/Fe–O in FLN-E-43 by Equation (7) are from 2.064 to 2.239 Å. The average bond length of Li/Fe-O in FLN-E-43 by Equation (7) is shorter than that of Li–O in LN43 by about 0.005 Å in the Li vacancy model. The long Li/Fe–O^4, 5, 6^ bond distance is shorter, but the short Li/Fe-O^7, 8, 9^ bond distance is longer in FLN-E-43. The discrepancy in values may be attributed to the hybrid calculation of bond lengths of Li–O and Fe–O. 

It should also be noted that the bond length of Li/Fe–Nb obviously decreased in Fe-doped LiNbO_3_. This reveals a tighter bonding in Fe–Nb than in Li–Nb. A similar conclusion would be derived from the observation of bond angles of O–Li/Fe–O and O–Nb–O. The bond angles of O^4^–Nb–O^5^ and O^4^–Li/Fe–O^5^, facing the plane-connected NbO_6_ and LiO_6_ frameworks, stretch out to make the distance between Li/Fe and Nb shorter. Moreover, the bond angles of O^7^–Li/Fe–O^8^ and O^1^–Nb–O^2^, which both face the plane connected the vacancy, bend along the *xy* plane, inducing shrinkage in the *xy* direction. The rest of the bond angles of O–Li/Fe–O and O–Nb–O stretch out or bend, resulting in an expansion in the c axis. This reveals that NbO_6_ and LiO_6_ frameworks are distorted by the doping Fe ion.

On the other hand, the lattice parameters of the Li vacancy model with Fe^+2^ ions calculated by Equation (9) are slightly larger than in the Li vacancy model with Fe^+2^ ions, calculated by Equation (7); the bond lengths of Li/Fe-O are from 2.065 to 2.239 Å and the position of Li ion is lower in the model by Equation (9) than by Equation (7). The reported value of Fe^+2^-O bond distances is about 2.08 to 2.18, which is higher than that of Fe^+3^-O and related to the lattice relaxation, while the valence is changed from Fe^+2^ to Fe^+3^ [31,32]. Our results show the same trend. Consequently, there are no obvious conflicts in the Li vacancy models with Fe^+2^ or Fe^+3^ ions to describe the defect model of Fe-doped LiNbO_3_.

Considering the impact of the pushed-back Nb* ion and the elimination of VNb at the regular Nb site, there is an obvious lattice distortion at the inter-plane-connected NbO_6_ and LiO_6_ framework. The bond angles of O^4^–Nb–O^5^, facing the plane connected the NbO_6_ and LiO_6_ framework, stretch out to contain the pushed-back Nb* ion. The increment of the bond lengths of Li/Fe–O^4, 5, 6^ and Li/Fe–Nb may be due to the Coulomb repulsion, accompanied by shortening bond lengths of Li/Fe–O^7, 8, 9^ and stretching bond angles of O^7^–Li/Fe–O^8^ due to the vanishing of VNb. The decrement of the distance of Li/Fe–vacancy–Nb could also be observed, which is related to the displacement of Li and Nb in the c axis. This coincides with the observation of A1 (TO1) at Raman spectra. Therefore, the speculation about doping Fe ions in LinbO_3_ accompanied by pushing Nb* ions back to the regular Nb site is possible.

## 4. Conclusions 

Various analyses were employed to investigate the defect structure of congruent LiNbO_3_ and Fe-doped LiNbO_3_ with *x* mol % Fe (*x* = 0.57–3.3) synthesized via the combustion method. 

The molar ratio of [Li]/([Li]+[Nb]) of our sample determined by *T*_c_ via DSC shows two values of 46.55% and 49.24% in LN43. Fe/Nb ratio of Fe-doped LiNbO_3_ derived EPMA analysis is from 0.57% to 3.314%. Two *T*_c_ peaks were observed in DSC due to phase splitting [36], and the phase at lower *T*_c_ disappears with an increase in doping Fe concentration, which reveals that congruent LiNbO_3_ and Fe-doped LiNbO_3_ synthesized via the combustion method have lots of intrinsic defects. 

From the analysis of XPS, divalent and trivalent Fe ions coexist at the regular Li site in the lattice structure of LiNbO_3_. The existence of an OH bond reveals the combination of the proton with an O ion near the vacancy defect, which is due to the charge compensation when the Li ion was replaced by the Nb* ion. The UV-vis spectra show the evidence of Fe^+2^ in Fe-doped LiNbO_3_; the redshift of absorption edge implies that Fe ions replace Li ions at the regular Li site. By way of inspecting the Raman spectra, doping with Fe causes O–Li–O and O–Nb–O stretching and bending, which give rise to the deformation of the NbO_6_ and LiO_6_ framework. In addition, Raman spectra exhibits the displacements along the c axis of the Li and Nb ions, which implies that Fe ions not only substitute for Li but push Nb* ions back to the regular Nb site.

According to the controversy about the defect model of LiNbO_3_ and the dispute about the occupation site of Fe, several models for comparison were adopted in the Rietveld refinement of powder X-ray diffraction collected by synchrotron radiation. The goodness of fit parameters for the fit, like χ^2^, *R*_wp_, *R*_p_, and *R*_(f_^2^_)_, the isotropic displacement parameters, Uiso, and corresponding refined atomic position, bond lengths, and bond angles were used to evaluated the fitting results. This reveals that the fit result of congruent LiNbO_3_ in the Nb vacancy model leads to an improbably distorted structure. The mixed-vacancy model and Li vacancy model are appropriate for interpreting the structures of congruent LiNbO_3_ synthesized by the combustion method. 

The doping models for Fe-doped LiNbO_3_ are rewritten based on the mixed-vacancy model and Li vacancy model and the doping mechanism such as the substitution of Li ion only or both Li and Nb* ion by the different oxidation state of Fe ion were considered in these models. The dispute about the Nb* ions being pushed back to the regular Nb site was also taken into account.

The refinement data of PXRD reveal that doping Fe ions in LiNbO_3_ causes lattice shrinkage and a slight distortion of the NbO_6_ and LiO_6_ framework. Li ions shift slightly upward and the average bond length of Li–O is shorter by about 0.005 Å in Fe-doped LiNbO_3_. The decreased bond length of Li/Fe–Nb shows a tighter bonding in Fe–Nb than in Li–Nb. The bending of bond angles of O–Li/Fe–O and O–Nb–O induces a shrinkage in the *xy* axis and the stretching bond angles of O–Li/Fe–O and O–Nb–O result in an expansion in the c axis. The pushed-back Nb* ion and the elimination of VNb causes an obvious decrement of the distance of Li/Fe-vacancy-Nb, related to the displacement of Li and Nb in the c axis. This is coincident with the observation of A1 (TO1) at Raman spectra. Therefore, speculation about doping Fe ions in LinbO_3_ at the same time as pushing Nb* ions back to the regular Nb site is possible. The Li vacancy model with Fe^+2^ ions is also reasonable and the lattice shrinkage is smaller than in the model with Fe^+3^ ions. We conjecture that the defect structure of Fe-doped LiNbO_3_ contains Li and Nb vacancies; Fe^+2^/Fe^+3^ ions are substituted for the Li ions at the regular Li site and may push the Nb* ion back to the regular Nb site.

## Figures and Tables

**Table 1:** The Currie temperature of congruent LiNbO_3_ (LN41 to LN43) and Fe-doped LiNbO_3_ (FLN-*x*-43, *x* = A to E) by DSC measurement and the corresponding *X*_c_ by Equation (4).

Sample	*T*_c1_ (°C)	*X*_c_ (%)	*T*_c2_ (°C)	*X*_c_ (%)
LN41	1169.48	49.17	1066.31	46.54
LN42	1172.84	49.25	1067.89	46.58
LN43	1172.31	49.24	1066.58	46.55
FLN-A-43	1171.86	-	1066.43	-
FLN-B-43	1171.24	-	1061.23	-
FLN-C-43	1170.01	-	-	-
FLN-D-43	1171.86	-	-	-
FLN-E-43	1170.00	-	-	-

**Table 2:** Fe/Nb ratio of Fe-doped LiNbO_3_ (FLN-*x*-43, *x* = A to E) by EPMA analysis.

Sample	Fe/Nb (mol %)
FLN-A-43	0.560
FLN-B-43	0.765
FLN-C-43	1.299
FLN-D-43	1.856
FLN-E-43	3.314

**Table 3:** Refined lattice parameters and agreement factors of LN43 and FLN-E-43 in the different models obtained from the Rietveld refinement. The errors represent the standard deviation (statistical only).

**LN43**	**Results of Iyi et al. [17]**		**Nb Vacancy Model**		**Mixed-Vacancy Model**		**Li Vacancy Model**	
**Formula**	[Li_0.951_Nb*_0.0098_] [Nb]O_3_		[Li_0.9748_Nb*_0.0252_] [Nb_0.9798_]O_3_		[Li_0.9748_Nb*_0.0101_] [Nb_0.9950_]O_3_		[Li_0.9748_Nb*_0.0050_] [Nb]O_3_	
a (Å)	5.1499 (1)		5.14643 (3)		5.14643 (1)		5.14642 (8)	
c (Å)	13.8647 (4)		13.84307 (4)		13.84306 (6)		13.84305 (8)	
volume (Å^3^)	318.45		317.52 (3)		317.52 (3)		317.52 (2)	
Uiso_Li/Nb*_	0.0134 (3)		0.0543 (2)		0.0200 (3)		0.0096 (2)	
Uiso_Nb_	0.0053 (7)		0.0063 (9)		0.0062 (1)		0.0061 (5)	
Uiso_O_	0.0069 (3)		0.0012 (4)		−0.0007 (4)		−0.0013 (6)	
χ^2^	1.924		1.075		1.021		1.009	
*R*_wp_	0.05		0.0686		0.0668		0.0664	
*R*_p_	0.0382		0.0464		0.0445		0.0440	
*R*_(f_^2^_)_	0.0168		0.0400		0.0355		0.0342	
**FLN-E-43**	**Model by Equation (5)**	**Model by Equation (6)**		**Model by Equation (7)**		**Model by Equation (8)**		**Model by Equation (9)**
**Formula**	[Li_0.9415_Fe_0.0333_ Nb*_0.0101_][Nb_0.9950_]O_3_	[Li_0.9415_Fe_0.0333_ Nb*_0.0076_][Nb_0.9975_]O_3_		[Li_0.9415_Fe_0.0333_ Nb*_0.0050_][Nb]O_3_		**[Li_0.9263_Fe_0.0330_ ][Nb_0.9950_]O_3_**		[Li_0.9415_ Fe_0.0333_ Nb*_0.0050_][Nb]O_3_
a (Å)	5.14605 (0)	5.14604 (7)		5.14604 (3)		5.14603 (2)		5.14604 (4)
c (Å)	13.84494 (0)	13.84492 (5)		13.84491 (1)		13.84486 (4)		13.84491 (5)
volume (Å^3^)	317.51 (9)	317.51 (8)		317.51 (7)		317.51 (5)		317.51 (8)
Uiso_Li/Nb*/Fe_	0.0226 (5)	0.0183 (0)		0.0140 (3)		0.0050 (7)		0.0144 (1)
Uiso_Nb_	0.0071 (7)	0.0071 (5)		0.0071 (2)		0.0070 (1)		0.0071 (3)
Uiso_O_	0.0010 (8)	0.0007 (5)		0.0004 (3)		0.0011 (6)		0.0004 (2)
χ^2^	1.377	1.368		1.361		1.343		1.363
*R*_wp_	0.0788	0.0786		0.0784		0.0778		0.0784
*R*_p_	0.0562	0.0560		0.0558		0.0555		0.0558
*R*_(f_^2^_)_	0.0331	0.0324		0.0316		0.0297		0.0318

**Table 4:** Refined atomic position of LN43 and FLN-E-43 in the different models obtained from the Rietveld refinement. The errors represent the standard deviation (statistical only).

Atom Type	Site	Model	x	y	z
Li/Nb*/Fe	6a	Results of Iyi et al. [15]	0	0	0.2809 (0)
		LN43 in Nb vacancy model	0	0	0.2828 (9)
		LN43 in the mixed-vacancy model	0	0	0.2809 (8)
		LN43 in Li vacancy model	0	0	0.2803 (8)
		FLN-E-43 in Equation (5)	0	0	0.2838 (8)
		FLN-E-43 in Equation (6)	0	0	0.2836 (8)
		FLN-E-43 in Equation (7)	0	0	0.2833 (8)
		FLN-E-43 in Equation (8)	0	0	0.2828 (7)
		FLN-E-43 in Equation (9)	0	0	0.2834 (8)
Nb	6a	Results of Iyi et al. [15]	0	0	0
		LN43 in Nb vacancy model	0	0	0
		LN43 in the mixed-vacancy model	0	0	0
		LN43 in Li vacancy model	0	0	0
		FLN-E-43 in Equation (5)	0	0	0
		FLN-E-43 in Equation (6)	0	0	0
		FLN-E-43 in Equation (7)	0	0	0
		FLN-E-43 in Equation (8)	0	0	0
		FLN-E-43 in Equation (9)	0	0	0
O	18b	Results of Iyi et al. [15]	0.0481 (0)	0.3433 (0)	0.0638 (0)
		LN43 in Nb vacancy model	0.0476 (7)	0.3432 (11)	0.06391 (25)
		LN43 in the mixed-vacancy model	0.0467 (7)	0.3429 (11)	0.0639790 (0)
		LN43 in Li vacancy model	0.0464 (7)	0.3427 (11)	0.06403 (24)
		FLN-E-43 in Equation (5)	0.0475 (9)	0.3427 (14)	0.06462 (31)
		FLN-E-43 in Equation (6)	0.0473 (9)	0.3426 (14)	0.06466 (31)
		FLN-E-43 in Equation (7)	0.0471 (9)	0.3426 (14)	0.06468 (31)
		FLN-E-43 in Equation (8)	0.0468 (9)	0.3424 (14)	0.06474 (31)
		FLN-E-43 in Equation (9)	0.0472 (9)	0.3426 (14)	0.06467 (31)

**Table 5:** Refined bond lengths, and bond angles of LN43 in the different models obtained from the Rietveld refinement. The errors represent the standard deviation (statistical only).

LN43	Results of Iyi et al. [15]	Nb Vacancy Model	Mixed-vacancy Model	Li Vacancy Model
Bond length (Å)^a^				
Nb-O^1,2,3^	1.879 (2)	1.879 (5)	1.879 (5)	1.880 (5)
Nb-O^4,5,6^	2.126 (3)	2.123 (4)	2.125 (4)	2.125 (4)
Li-O^4,5,6^	2.254 (5)	2.236 (10)	2.257 (9)	2.264 (9)
Li-O^7,8,9^	2.061 (3)	2.066 (5)	2.053 (4)	2.049 (4)
Li-Nb	3.038 (6)	3.007 (13)	3.034 (11)	3.042 (11)
	3.0609 (14)	3.0527 (29)	3.0588 (26)	3.0607 (26)
	3.369 (3)	3.378 (6)	3.366 (5)	3.362 (5)
Distance of Li-vacancy-Nb	3.895 (6)	3.91600 (7)	3.88962 (6)	3.88131 (7)
Bond Angle (˚)				
O^1^-Nb-O^2^	99.65 (11)	99.64 (14)	99.60 (13)	99.58 (13)
O^4^-Nb-O^5^	79.91 (9)	80.03 (16)	80.14 (15)	80.19 (15)
O^2^-Nb-O^5^	165.91 (12)	166.00 (21)	166.10 (21)	166.15 (21)
O^1^-Nb-O^5^	90.06 (12)	90.01 (9)	89.96 (9)	89.95 (9)
O^3^-Nb-O^5^	88.65 (10)	88.61 (8)	88.61 (8)	88.61 (8)
O^4^-Li-O^5^	74.54 (17)	75.3 (4)	74.62 (35)	74.41 (34)
O^7^-Li-O^8^	109.11 (17)	108.5 (4)	109.15 (31)	109.37 (30)
O^5^-Li-O^8^	153.5 (3)	154.6 (6)	153.5 (5)	153.2 (5)
O^5^-Li-O^7^	80.91 (12)	81.16 (13)	80.97 (13)	80.90 (12)
O^5^-Li-O^9^	89.48 (11)	89.67 (16)	89.28 (16)	89.15 (15)

^a^ The corresponding O atom in the lattice is represented in Figure 4.

**Table 6:** Refined bond lengths, and bond angles of FLN-E-43 in the different models obtained from the Rietveld refinement. The errors represent the standard deviation (statistical only).

FLN-E-43	Model by Equation (5)	Model by Equation (6)	Model by Equation (7)	Model by Equation (8)	Model by Equation (9)
Bond length (Å)^a^					
Nb-O^1,2,3^	1.881 (6)	1.882 (6)	1.882 (6)	1.882 (6)	1.882 (6)
Nb-O^4,5,6^	2.118 (5)	2.118 (5)	2.119 (5)	2.119 (5)	2.118 (5)
Li/Fe-O^4,5,6^	2.233 (9)	2.237 (9)	2.239 (9)	2.246 (9)	2.239 (9)
Li/Fe-O^7,8,9^	2.068 (5)	2.066 (5)	2.064 (5)	2.060 (5)	2.065 (5)
Li/Fe-Nb	2.993 (11)	2.997 (11)	3.000 (10)	3.007 (10)	2.999 (11)
	3.0491 (24)	3.0499 (24)	3.0506 (24)	3.0523 (23)	3.0504 (24)
	3.385 (5)	3.383 (5)	3.382 (5)	3.378 (5)	3.382 (5)
Distance of Li/Fe-vacancy-Nb	3.93030 (1)	3.92752 (7)	3.92337 (0)	3.91629 (7)	3.92198 (4)
Bond Angle (˚)					
O^1^-Nb-O^2^	99.25 (17)	99.24 (17)	99.22 (17)	99.19 (17)	99.23 (17)
O^4^-Nb-O^5^	80.37 (20)	80.40 (20)	80.43 (20)	80.48 (20)	80.42 (20)
O^2^-Nb-O^5^	166.53 (27)	166.56 (27)	166.59 (27)	166.65 (27)	166.58 (27)
O^1^-Nb-O^5^	90.06 (11)	90.06 (11)	90.05 (11)	90.03 (11)	90.05 (11)
O^3^-Nb-O^5^	88.74 (10)	88.74 (10)	88.74 (10)	88.74 (10)	88.74 (10)
O^4^-Li/Fe-O^5^	75.5 (4)	75.4 (4)	75.3 (4)	75.1 (4)	75.3 (4)
O^7^-Li/Fe-O^8^	108.33 (30)	108.43 (31)	108.52 (30)	108.73 (30)	108.49 (30)
O^5^-Li/Fe-O^8^	154.8 (5)	154.6 (5)	154.5 (5)	154.2 (5)	154.6 (5)
O^5^-Li/Fe-O^7^	81.20 (12)	81.17 (12)	81.15 (12)	81.10 (12)	81.15 (12)
O^5^-Li/Fe-O^9^	89.70 (16)	89.64 (16)	89.59 (16)	89.46 (16)	89.60 (16)

**^a^** The corresponding O atom in the lattice is represented in Figure 4.
